# Celiac disease symptom profiles and their relationship to gluten-free diet adherence, mental health, and quality of life

**DOI:** 10.1186/s12876-023-03101-x

**Published:** 2024-01-02

**Authors:** Cara Dochat, Niloofar Afari, Rose-Marie Satherley, Shayna Coburn, Julia F. McBeth

**Affiliations:** 1 San Diego State University/University of California San Diego Joint Doctoral Program in Clinical Psychology, San Diego, CA USA; 2 VA San Diego Healthcare System, San Diego, CA USA; 3 University of California San Diego, La Jolla, CA USA; 4 Department of Psychological Interventions, University of Surrey, Guildford, UK; 5 Children’s National Health System, Washington, DC USA; 6 George Washington University School of Medicine & Health Sciences, Washington, DC USA; 7 Celiac Disease Foundation, Woodland Hills, CA USA

**Keywords:** Celiac Disease, Quality of life, Gluten-free diet, Latent profile analysis

## Abstract

**Background:**

A subgroup of adults with celiac disease experience persistent gastrointestinal and extraintestinal symptoms, which vary between individuals and the cause(s) for which are often unclear.

**Methods:**

The present observational study sought to elucidate patterns of persistent symptoms and the relationship between those patterns and gluten-free diet adherence, psychiatric symptoms, and various aspects of quality of life (QOL) in an online sample of adults with celiac disease. U.S. adults with self-reported, biopsy-confirmed celiac disease (*N* = 523; *M*age = 40.3 years; 88% women; 93.5% White) voluntarily completed questionnaires as part of the iCureCeliac® research network: (a) Celiac Symptoms Index (CSI) for physical symptoms and subjective health; (b) Celiac Dietary Adherence Test for gluten-free diet adherence; (c) PROMIS-29, SF-36, and Celiac Disease Quality of Life Survey for psychiatric symptoms and QOL. Symptom profiles were derived using latent profile analysis and profile differences were examined using auxiliary analyses.

**Results:**

Latent profile analysis of CSI items determined a four-profile solution fit best. Profiles were characterized by: (1) little to no symptoms and excellent subjective health (37% of sample); (2) infrequent symptoms and good subjective health (33%); (3) occasional symptoms and fair to poor subjective health (24%); (4) frequent to constant symptoms and fair to poor subjective health (6%). Profiles 2 and 3 reported moderate overall symptomology though Profile 2 reported relatively greater extraintestinal symptoms and Profile 3 reported relatively greater gastrointestinal symptoms, physical pain, and worse subjective health. Profiles differed on anxiety and depression symptoms, limitations due to physical and emotional health, social functioning, and sleep, but not clinical characteristics, gluten-free diet adherence, or QOL. Despite Profile 3’s moderate symptom burden and low subjective health as reported on the CSI, Profile 3 reported the lowest psychiatric symptoms and highest quality of life on standardized measures.

**Conclusions:**

Adults with celiac disease reported variable patterns of persistent symptoms, symptom severity, and subjective health. Lack of profile differences in gluten-free diet adherence suggests that adjunctive dietary or medical assessment and intervention may be warranted. Lower persistent symptom burden did not necessarily translate to better mental health and QOL, suggesting that behavioral intervention may be helpful even for those with lower celiac symptom burden.

**Supplementary Information:**

The online version contains supplementary material available at 10.1186/s12876-023-03101-x.

Celiac disease, an autoimmune condition, affects 48–300 million people worldwide [1, 2]. For individuals with celiac disease, ingestion of gluten prompts an autoimmune response, damaging the structure and function of the small intestine and causing symptoms like headache, fatigue, skin manifestations, and neurologic symptoms [3–5]. Managing celiac disease requires adherence to a strict gluten-free diet (GFD), which supports intestinal recovery and symptom relief for the majority [6].

Despite optimal GFD adherence, 20–40% of adult patients continue to experience symptoms, and the cause for persisting symptoms is often unclear [4, 7–14]. In a study including 99 U.S. adults with persistent symptoms on a GFD, the most common causes included ongoing gluten exposure (36%), co-occurring irritable bowel syndrome (IBS; 22%), and refractory celiac disease (13%), [11], a finding replicated in 140 adult patients in Italy [15]. Complete gluten removal from one’s diet may not be achievable, and even small amounts of gluten exposure can contribute to persistent symptoms and incomplete intestinal recovery [16, 17]. Alternatively, persistent symptoms may indicate the presence of other food sensitivities or other medical conditions such as IBS, characterized by specific gastrointestinal symptoms (abdominal pain and bloating, painful bowel movements, and diarrhea and/or constipation) [18, 19]. Women [20]. and those with fewer years since diagnosis [8] may be more prone to persistent symptoms.

Persistent symptoms in celiac disease are associated with worse physical functioning, impaired quality of life, and greater likelihood of anxiety and depression [8, 12–14, 21, 22]. Furthermore, the severity of ongoing gastrointestinal symptoms [23] is associated with reduced quality of life across specific domains (e.g., social functioning), greater anxiety, and depression [14]. While a GFD helps some with anxiety and depression, for others, these symptoms persist or emerge even after intestinal recovery. The connections between these psychiatric symptoms and persistent physical symptoms is not well understood [26].

Regardless of the underlying nature, persistent symptoms pose challenges in celiac disease management. Examining specific patterns of these symptoms and their associations with relevant clinical variables including disease factors, GFD adherence, psychiatric symptoms, and quality of life can offer insights into differential diagnosis and optimizing treatment. To date, no study has examined patterns of persistent gastrointestinal and extraintestinal symptoms and their relationships to these variables. The aims of this study were to use data available from the CureCeliac® research network to: [1] identify patterns of persistent symptoms and subjective health ratings among U.S. adults with celiac disease; and [2] examine whether persistent symptom profile groups report differences in GFD adherence, psychiatric symptoms, and quality of life and functioning.

## Methods

### Design and participants

A cross-sectional survey was administered between April 2019 and May 2020 as part of the iCureCeliac® research network registry hosted by the Celiac Disease Foundation. Participants with celiac disease are self-referred to participate in the registry on a rolling basis through the Celiac Disease Foundation’s website and email newsletter. The registry was launched in February 2016 as a Patient-Centered Outcomes Research Institute (PCORI)-funded project and developed in collaboration with the University of Southern California, the Celiac Disease Foundation Medical Advisory Board, and other members of the celiac disease scientific and medical communities. All participants provided informed consent before survey initiation. De-identified data from registry participants were included from adults (≥ 18 years old) with a self-reported celiac disease diagnosis made via intestinal biopsy, serology (blood test), or genetic testing, who reported their country of origin as the U.S. The Celiac Symptom Index was completed by *N* = 523. Complete data on all other measures were provided by *n* = 317.

### Measures

#### Sociodemographic variables, disease factors, and co-occurring conditions

Participants self-reported sociodemographic, health, and disease factor information, including current age, gender, race/ethnicity, household income, educational attainment, age at celiac disease diagnosis, diagnostic method, diagnostic reason, and co-occurring physical and mental health conditions.

#### Celiac symptoms and subjective health

The Celiac Symptom Index (CSI) [27] is a 16-item self-report instrument assessing specific celiac symptoms and subjective health in the past four weeks. Twelve items assess specific symptoms rated from 1 (*none of the time*) to 5 (*all of the time*). Four items assess subjective aspects of physical health, including subjective rating of celiac-specific health and general health, rated from 1 (*excellent*) to 5 (*terrible*), and subjective rating of comfort and one’s health compared to the health of others, rated from 1 (*strongly agree*) to 5 (*strongly disagree*). Item ratings are summed to create a total score. Higher scores indicate greater symptom burden and lower subjective health. Internal consistency reliability for CSI total scores were good in the development sample (α = 0.88) and current sample (α = 0.85).

#### Gluten-free diet adherence

The Celiac Dietary Adherence Test (CDAT) [28] is a 7-item self-report measure of GFD adherence. Items assess low energy, headaches, ability to follow a GFD while dining out, carefully considering consequences of one’s behavior, perception of oneself as a failure, perceived impact of accidental gluten exposure on health, and number of intentional gluten exposures in the past four weeks. Item ratings are summed to create a total score. Lower scores indicate greater gluten-free adherence. CDAT scores are highly correlated with standardized dietitian evaluation and biomarkers of celiac disease-linked antibodies. Receiver operating characteristic curve analysis in the development sample showed that CDAT scores of < 13 indicate good adherence, scores of 13–17 indicate moderate adherence, and scores > 17 indicate poor adherence [28]. Internal consistency reliability in the current sample was low (α = 0.57) and not published for the development sample.

#### General health-related quality of life and functioning

The PROMIS-29 [29] is a 29-item self-report instrument assessing seven domains of health-related quality of life in the past seven days: depression (4 items), anxiety (4 items), physical function (4 items), fatigue (4 items), sleep disturbance (4 items), and ability to participate in social roles and activities (4 items). A final item assessing pain intensity was not included in the present analyses. Raw scores are converted to *t*-scores for all scales. Higher *t*-scores indicate more of the domain being assessed (e.g., higher physical functioning or greater fatigue). PROMIS scales have strong psychometric properties [29].

The RAND 36-Item Health Survey version 1.0 (SF-36) [30] is a 36-item self-report instrument assessing eight domains of health-related quality of life in the past four weeks: physical functioning (10 items), social functioning (2 items), role limitations due to physical functioning (4 items), role limitations due to emotional problems (3 items), energy/fatigue (4 items), emotional well-being (5 items), general health (5 items), and pain (2 items). Item ratings are transformed to scaled scores and averaged within each domain to provide eight scores between 0 and 100. Higher scores on each scale indicate better health-related quality of life. The SF-36 has demonstrated reliability and validity across multiple chronic illness populations, and has been used in celiac disease [31]. Internal consistency reliability of SF-36 scales in the current sample was high (α range = 0.83-0.92; ω range = 0.83-0.93).

#### Anxiety and depression symptoms

The 4-item short forms of the PROMIS anxiety and depression scales [29] were extracted from the PROMIS-29 to assess the frequency of anxiety and depression symptoms in the past seven days. Raw scores are converted to *t*-scores for both scales. Higher *t*-scores indicate greater symptomology. PROMIS scales have strong psychometric properties [29]. Internal consistency reliability in the current sample was excellent for both anxiety (α = 0.90; ω = 0.90) and depression (α = 0.93; ω = 0.93).

#### Celiac disease-specific quality of life

The Celiac Disease Quality of Life Survey (CD-QOL) [32] is a 20-item self-report instrument assessing celiac disease-specific quality of life in the past 30 days. One item is reverse coded and item ratings are summed to create total and subscale scores: limitations (9 items), dysphoria (4 items), health concerns (5 items), and inadequate treatment (2 items). Higher scores indicate lower celiac disease-specific quality of life. Internal consistency reliability of CD-QOL total score was excellent (α=0.92; ω=0.92) and subscale scores were acceptable (α range = 0.83-0.88; ω range = 0.83-0.88) in the current sample, and not published for the development sample.

### Statistical analyses

Latent profile analysis (LPA) was used to identify celiac disease health profiles using CSI items as indicators. CSI items 1–11 and 14 assess specific symptom severity in the past four weeks, and items 12, 13, 15, and 16 assess subjective ratings of health with no timeframe specified. LPA was conducted on both the total sample (*N* = 523) and subsample with complete data on all measures (*n* = 317) in MPlus version 8 [33]. Successive latent profile models were fit, increasing the number of potential profiles by one until model fit was not significantly improved. Comparative model fit was evaluated using the bootstrapped likelihood ratio test (BLRT) [34] and Lo-Mendell-Rubin adjusted likelihood ratio test (LMRT) [35], where a *p-*value of < 0.05 indicates better fit than a hypothetical model with one fewer profile [36]. Comparative model fit was also evaluated using Akaike Information Criterion (AIC) [37], Bayesian Information Criterion (BIC) [38], and sample size-adjusted BIC (s-BIC) [39], where lower values indicate better model fit. Probabilities of group classification (posterior classification probabilities) were examined for all competing models, with average probabilities ≥ 0.70 indicating an appropriate profile solution [40]. Entropy, a classification accuracy metric, was also examined. Higher entropy (preferably > 0.80) [41] demonstrates greater classification accuracy. Latent profiles were interpreted using conditional response means and latent profile probabilities.

Based on posterior classification probabilities, individuals were assigned to profile groups. Potential profile group differences were then examined for sociodemographic characteristics, disease factors, and observed outcomes (questionnaire scores) using the BCH method (AUXILIARY function) in MPlus [42–45]. This method accounts for uncertainty in individual profile membership and provides a chi-squared test of profile differences as well as pairwise comparisons. Chi-squared tests and pairwise comparisons were considered statistically significant at *p* < .05. Because the CSI and CDAT have two overlapping items (“Have you been bothered by low energy level during the past 4 weeks?” and “Have you been bothered by headaches during the past 4 weeks?”), AUXILIARY analyses were conducted for CDAT total score (7 items) and CDAT total score minus overlapping items (5 items).

## Results

### Sample characteristics

Sample characteristics are shown in Table 1. Most participants identified as women (88%) and white (92%). Current age ranged from 18 to 83 years (*M* = 41, *SD =* 15). Age at celiac disease diagnosis ranged from 2 to 82 years (*M* = 35, *SD =* 15). Years since diagnosis ranged from 0 to 78 (*M* = 6, *SD* = 8), with 8% within 1 year of diagnosis, 25% within 2 years of diagnosis, and 50% within 3 years of diagnosis. Mean sample CDAT score suggested good to moderate GFD adherence. Specifically, 45% reported good adherence, 43% reported moderate adherence, and 12% reported poor adherence. Sample mean anxiety and depression symptom *t*-scores were within normal range of the U.S. population. Approximately half of the sample reported lifetime diagnosis of a mental health condition and a quarter reported significantly elevated (*t* ≥ 60) anxiety and depression symptoms at present.

**Table 1:** Sociodemographic Variables, Disease Factors, and Mean Questionnaire Scores for Total Sample (N = 523) and Subsample with Complete Data (*n* = 317)

Measure	Total (*N* = 523)	Subsample (*n* = 317)
**Sociodemographic Variables and Disease Factors**		
Age, *M* (*SD*)	40.26 (14.94)	40.99 (15.13)
Female	88.0%	87.7%
Race/Ethnicity		
White	93.5%	92.1%
Hispanic/Latinx	3.1%	3.5%
American Indian/Alaskan Native	1.9%	2.5%
Black	0.6%	1.0%
Asian	0.4%	0.3%
Native Hawaiian/Pacific Islander	0.2%	0.0%
Other	0.4%	0.6%
Household Income^†^		
Less than $50,000	--	17.0%
$50,000-$100,000	--	26.5%
$100,000-$200,000	--	21.5%
$200,000 or more	--	6.7%
Missing data		28.1%
Education^**‡**^		
High School Diploma	--	3.8%
Vocational, Trade, or Associate’s degree	--	12.3%
Bachelor’s degree or some college	--	47.7%
Professional, Master’s, or Doctorate degree	--	23.0%
Missing data	--	12.3%
Age at diagnosis, *M* (*SD*)	34.19 (15.19)	35.02 (15.06)
Years since diagnosis, *M* (*SD*)	6.00 (8.01)	5.91 (7.47)
Diagnostic method		
Biopsy (small bowel/intestine)	81.0%	83.0%
Serology/blood test	17.3%	14.2%
Other	1.7%	1.5%
Diagnostic reason		
Symptomatic	75.0%	76.7%
Other	25.0%	23.3%
Co-occurring Conditions		
Lifetime diagnosis of any mental health condition	53.2%	52.5%
Lifetime diagnosis of depressive disorder	35.4%	35.0%
Lifetime diagnosis of anxiety disorder	40.2%	40.4%
Bone or joint pain (current)	--	52.1%
Weight gain or loss (current)	--	42.0%
Fibromyalgia or muscle pain (current)	--	31.2%
Peripheral neuropathy (current)	--	30.9%
Irritable bowel syndrome (diagnosed at any time)	--	29.7%
Irritable bowel syndrome diagnosed prior to CeD		23.3%
Irritable bowel syndrome diagnosed after CeD		8.2%
Alopecia/Hair loss (current)	--	28.4%
Lactose intolerance (current)	--	27.8%
Thyroid disease (diagnosed at any time)	--	24.9%
Dermatitis herpetiformis (current)	--	24.0%
Arthritis (excluding Rheumatoid arthritis) (current)	--	23.0%
Menstrual irregularities (women only) (current)	--	21.9%
Eczema (current)	--	21.1%
Osteopenia or osteoporosis (current)	--	14.2%
Psoriasis (diagnosed at any time)	--	8.8%
Rheumatoid arthritis (diagnosed at any time)	--	7.3%
Ulcerative Colitis (diagnosed at any time)	--	2.8%
Type 1 diabetes mellitus (diagnosed at any time)	--	1.6%
Crohn’s disease (diagnosed at any time)	--	0.9%
Measures	*M* (*SD*)	*M* (*SD*)
CSI total	39.66 (9.89)	39.79 (9.89)
CDAT total	13.34 (3.69)	13.32 (3.58)
PROMIS-29 Anxiety	--	51.97 (9.51)
PROMIS-29 Depression	--	54.42 (9.66)
PROMIS-29 Pain Interference	--	52.51 (9.33)
PROMIS-29 Physical Function	--	49.20 (8.23)
PROMIS-29 Social Roles/Activities	--	49.77 (9.56)
PROMIS-29 Fatigue	--	57.79 (11.39)
PROMIS-29 Sleep Disturbance	--	52.91 (8.28)
SF-36 Physical Functioning	--	81.37 (22.48)
SF-36 Role Limitations – Physical health	--	55.54 (42.83)
SF-36 Role Limitations – Emotional problems	--	56.15 (42.61)
SF-36 Energy/Fatigue	--	38.79 (23.99)
SF-36 Emotional Wellbeing	--	63.92 (19.80)
SF-36 Social Functioning	--	70.82 (26.01)
SF-36 Bodily Pain	--	61.14 (24.95)
SF-36 General Health	--	51.07 (23.82)
CD-QOL Total	63.07 (16.17)^§^	62.39 (16.15)
CD-QOL Limitations	29.77 (8.27)^§^	29.71 (8.27)
CD-QOL Dysphoria	9.45 (4.09)^§^	9.25 (4.03)
CD-QOL Health Concerns	17.10 (4.82)^§^	16.77 (4.87)
CD-QOL Inadequate Treatment	6.75 (2.11)^§^	6.66 (2.05)

*Note. M* = mean; *SD* = standard deviation; CSI = Celiac Symptom Index; CDAT = Celiac Dietary Adherence Test; PROMIS = Patient-Reported Outcomes Measurement Information System®; CD-QOL = Coeliac Disease Quality of Life Survey. All values are raw scores except for PROMIS measures, which are *t*-scores. Missing values indicate that data were not available for the full sample. Conditions with sample prevalence < 1% are not reported

^†^*n* = 228; values shown are percent out of *n* = 317 including missing data; valid percentages are: 23.7%, 36.8%, 29.8%, 9.6%

^**‡**^*n* = 279; values shown are percent out of *n* = 317 including missing data; valid percentages are: 1.1%, 4.3%, 14.0%, 15.5%, 38.8%, 2.9%, 18.3%, 5.0%

^§^*n* = 453

Most of the sample (96.5%) reported at least one comorbid physical health condition. The most common co-occurring conditions were pain-related, including bone or joint pain (52%), fibromyalgia or muscle pain (31%), peripheral neuropathy (31%), and arthritis (23% with non-specific arthritis; 7% with Rheumatoid arthritis). In terms of gastrointestinal conditions, 23% reported an IBS diagnosis prior to celiac disease diagnosis and 8% reported an IBS diagnosis made since celiac disease diagnosis. Smaller proportions reported a co-occurring inflammatory bowel disease such as ulcerative colitis (3%) and Crohn’s disease (1%). Current lactose intolerance was reported by 28% of participants. There were no differences in sociodemographic, disease factors, or questionnaire scores between the total sample and subsample with complete data on all measures (*p*s > 0.05; see Table 1).

### Latent profile analysis

#### Model Fit

Table 2 displays goodness-of-fit statistics for models with one to six profiles. A four-profile solution evidenced best fit and had adequate entropy and high posterior classification probabilities in both the total sample and subsample. The class proportions and conditional response means for the four-profile solution were nearly identical across the total sample and subsample. Thus, the four-profile solution was selected for interpretation and further analyses.

**Table 2:** Goodness-of-fit Statistics for Latent Profile Analysis Model Solutions (*N* = 317)

Profiles	Log Likelihood	AIC	BIC	s-BIC	Entropy	Smallest class %	LMRT *p*-value	LMRT meaning	BLRT *p*-value	BLRT meaning
1	-7735.392	15534.783	15655.068	15553.572	--	--	--	--	--	--
2	-7169.051	14436.102	14620.288	14464.871	0.899	44%	< 0.001	2 > 1	< 0.001	2 > 1
3	-7057.281	14246.562	14494.649	14285.313	0.850	21%	0.57	2 > 3	0.57	2 > 3
4	-6959.550	14085.101	14397.090	14133.833	0.882	6%	0.02	4 > 3	0.02	4 > 3
5	-6911.041	14022.082	14397.972	14080.796	0.876	5%	0.50	4 > 5	0.50	4 > 5
6	-6868.440	13970.880	14410.672	14039.575	0.892	2%	0.60	5 > 6	0.60	5 > 6

*Note*. AIC = Akaike information criterion; BIC = Bayesian information criterion; s-BIC = sample size-adjusted Bayesian information criterion; LMRT = Lo-Mendell-Rubin adjusted likelihood ratio test; BLRT = bootstrapped likelihood ratio test. All models tested using maximum likelihood estimation.

#### Profile characteristics

**Fig. 1:**
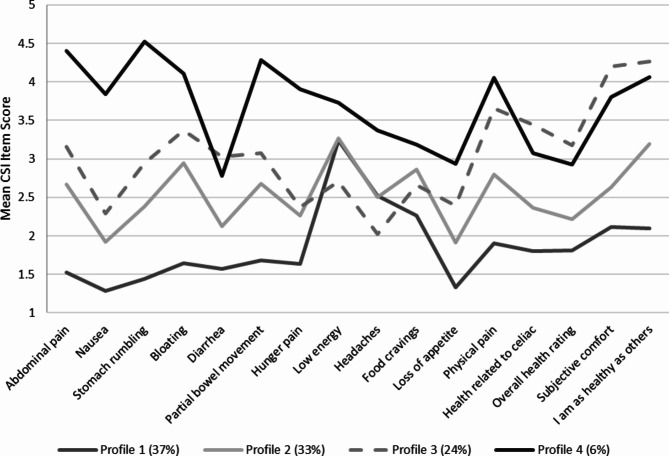
Conditional response means on CSI items for the LPA four-profile solution (*N* = 317). CSI = Celiac Symptoms Index. Higher item scores indicate greater symptomology and lower health ratings. Profile 1 is characterized by little to no symptoms and excellent subjective health, except for low energy. Profile 2 is characterized by more frequent symptoms than Profile 1, with a similar elevation on low energy and smaller elevations on bloating, food cravings, and physical pain. Participants in Profile 2 report good subjective health, and they neither agree nor disagree with statements about feeling comfortable or their health status compared to others. Profile 3 is characterized by more frequent symptoms than Profile 2 overall, except for low energy, headaches, and food cravings. Profile 3 shows elevations on most gastrointestinal symptoms and especially on physical pain. Those in Profile 3 report fair subjective health, low comfort, and poor health compared to others. Profile 4 is characterized by frequent to nearly constant symptoms, with notable elevations on all gastrointestinal symptoms, food cravings, low energy, headaches, and physical pain. Participants in Profile 4 reported fair subjective health, low comfort, and poor health compared to others

Conditional response means across the four profiles are shown in Fig. 1. Findings are summarized in Table 3. **Profile 1 (37%)** was characterized by little to no symptoms and excellent subjective health. Profile 1 showed relative elevations on low energy and headaches, which occurred on average “some of the time.” **Profile 2 (33%)** was characterized by more frequent symptoms than Profile 1, with relative elevations on low energy and smaller elevations on bloating, food cravings, and physical pain. Participants in Profile 2 reported good subjective health, and they neither agreed nor disagreed with statements about feeling comfortable or their health status compared to others. **Profile 3 (24%)** was characterized by more frequent symptoms than Profile 2 overall, except for low energy, headaches, and food cravings, which were higher in Profile 2. Profile 3 showed elevations on most gastrointestinal symptoms and especially on physical pain. Those in Profile 3 reported fair subjective health, low comfort, and poor health compared to others. **Profile 4 (6%)** was characterized by frequent to nearly constant symptoms, with notable elevations on all gastrointestinal symptoms, food cravings, low energy, headaches, and physical pain. Food cravings and headaches were relatively less frequent than other symptoms within Profile 4, but nevertheless more frequent than in other profiles. Participants in Profile 4 reported fair subjective health, low comfort, and poor health compared to others.

**Table 3:** Summarized results of latent profile analysis

Profile	Prominent symptoms	Subjective health ratings
**1 (37%)**	Low energy	Excellent
**2 (33%)**	Low energy, bloating, food cravings, physical pain	Good, feel as comfortable as others, similar health status to others
**3 (24%)**	Higher and a greater diversity of gastrointestinal symptoms overall, especially abdominal pain, bloating, diarrhea, partial bowel movement; physical pain	Fair, low comfort, poor health compared to others
**4 (6%)**	Highest gastrointestinal symptom burden overall, especially abdominal pain, nausea, stomach rumbling, bloating, partial bowel movement, and hunger pain; diarrhea rates similar to profile 3; higher extraintestinal symptoms than other profiles, including low energy, headaches, food cravings, loss of appetite, physical pain	Fair, low comfort, poor health compared to others

Profile 4 reported the greatest symptomology across both gastrointestinal and extraintestinal symptoms. Profiles 2 and 3 each reported moderate symptomology, where Profile 2 reported relatively greater extraintestinal symptomology and Profile 3 reported relatively greater gastrointestinal symptomology. Profile 1 reported lowest overall symptom burden, but nevertheless reported persistent low energy and headaches (comparable to or greater than in Profiles 2 and 3). In terms of specific gastrointestinal symptom frequency, Profiles 2 and 3 were both characterized by relative elevations on abdominal pain, bloating, and partial bowel movement, but only diarrhea was elevated in Profile 3. Nausea was prominent only in Profile 4.

### Auxiliary analyses

#### Sociodemographic variables

Summarized results of auxiliary analyses are shown in Table 4 and full results are shown in Supplement Table 1. No profile differences were found with regards to current age, sex, or race/ethnicity. Among those reporting education level (*n* = 279), participants in Profile 4 were more likely to have associates or trade school degrees and less likely to have master’s and doctoral degrees than those in Profiles 1 and 2. Among those reporting household income (*n* = 228), those in Profiles 1 and 2 were more likely to report incomes of $100K + and those in Profile 4 were more likely to incomes less than $50K. Given that no profile differences were detected for sociodemographic variables reported by the full subsample (*n* = 317), further auxiliary analyses were conducted without covariates.

**Table 4:** Summarized results of auxiliary analyses

Significant profile differences (*p* < .05)	No profile differences (*p* > .05)
Education level	Age
Household income level	Sex
Depression symptoms	Race (white v. other)
Emotional wellbeing	Age at diagnosis
Role limitations due to physical health	Years since diagnosis
Role limitations due to emotional health	Reason for diagnostic testing
Social functioning	Gluten-free diet adherence
Ability to participate in social roles	Anxiety symptoms (*p* = .08)
Energy/fatigue	Physical functioning
Fatigue	Body pain
Sleep disturbance	CD-QOL total
General health	CD-QOL limitations
	CD-QOL dysphoria
	CD-QOL health concerns
	CD-QOL inadequate treatment

*Note*. CD-QOL = Coeliac Disease Quality of Life Survey.

#### Disease factors

No profile differences were found regarding age at diagnosis, years since diagnosis, or reason for diagnostic assessment (i.e., symptomatic versus another reason).

#### Gluten-free diet adherence

Significant profile differences were found for GFD adherence when using the *CDAT total score*. Pairwise comparisons showed that adherence for Profile 3 was significantly greater than for Profiles 1, 2, and 4. However, when symptom items were removed from the CDAT score, there were no significant profile differences.

#### Anxiety and depression

Significant profile differences were found for *depression symptoms.* Marginally significant differences were found for *anxiety symptoms* (*p* = .08). Pairwise comparisons showed that anxiety and depression symptoms were significantly more severe for Profile 4 than Profiles 2 and 3, but not Profile 1.

#### General Health-Related Quality of Life and Functioning

Significant profile differences were found for SF-36 *role limitations due to physical health* and SF-36 *role limitations due to emotional health*. Pairwise comparisons showed that Profiles 1, 2, and 4 reported significantly greater role *limitations due to physical health* than Profile 3. Profile 4 reported significant greater role *limitations due to emotional health* than Profiles 1, 2, and 3.

Significant profile differences were found for SF-36 *emotional wellbeing*, where Profile 4 reported significantly worse *emotional wellbeing* than Profiles 1, 2, and 3. Additionally, Profile 3 reported significantly greater *emotional wellbeing* than Profile 1.

Significant profile differences were found for SF-36 *social functioning* and PROMIS-29 *ability to participate in social roles/activities*, where Profile 3 reported significantly greater social functioning and ability than Profiles 1 and 4. For PROMIS-29 *ability to participate in social roles/activities*, Profiles 1 and 2 were also significantly greater than Profile 4.

Significant profile differences were found for SF-36 *energy/fatigue* and PROMIS-29 *fatigue*, where Profile 4 reported significantly lower *energy* and higher *fatigue* than Profiles 1, 2, and 3. Additionally, Profile 3 reported significantly lower *fatigue* than Profiles 1 and 2. Perhaps relatedly, significant profile differences were found for PROMIS-29 *sleep disturbance*, where Profile 4 reported significantly greater *sleep disturbance* than Profiles 1, 2, and 3. Additionally, Profile 3 reported significantly less *sleep disturbance* than Profiles 1 and 2.

Significant profile differences were found for SF-36 *general health*, where Profile 3 reported significantly greater *general health* than Profiles 1, 2, and 4. Omnibus tests for profile differences were not significant for SF-36 *physical functioning*, PROMIS-29 *physical function*, SF-36 *bodily pain*, and PROMIS-29 *pain interference*.

#### Celiac disease-specific quality of life

No profile differences were found for CD-QOL total or subscale scores.

## Discussion

This study examined patterns of persistent symptoms and their relationships to disease management and wellbeing among U.S. adults with celiac disease. Four unique symptom profiles emerged. Profile 1, which comprised the largest proportion of the sample (37%), was characterized by overall low symptomology and excellent subjective health, but with persistent low energy and headaches. Profiles 2 and 3, the second (33%) and third (24%) largest, reported moderate overall symptomology, but differed from one another such that Profile 2 reported relatively greater extraintestinal symptomology and Profile 3 reported relatively greater gastrointestinal symptomology. Profile 4, the smallest profile (6%), was defined by the most severe symptomology across both extraintestinal and gastrointestinal symptoms, and was especially elevated in abdominal pain, nausea, stomach rumbling, bloating, partial bowel movement, and hunger pain compared to other profiles. Profile 4 was also consistently lowest in psychiatric wellbeing and various quality of life domains, consistent with literature showing that greater persistent gastrointestinal symptom burden relates to lower physical functioning, lower quality of life, and greater likelihood of anxiety and depression [8, 22, 46].

Most research to date has examined gastrointestinal symptom burden and its relation to quality of life [8, 22]. The present findings suggest that extraintestinal symptom burden may also impact quality of life and warrant intervention. Low energy, headaches, physical pain, and food cravings were present across all profiles, independent of gastrointestinal symptom severity. Research has shown that fatigue is common in adults with celiac disease and can persist despite GFD adherence [47, 48]. In the present study, greater persistent fatigue co-occurred with greater psychiatric symptoms and worse social functioning. Profile differences in sleep disturbance followed a similar pattern. It is possible that improving sleep quality through interdisciplinary intervention approaches (e.g., cognitive-behavioral therapy for insomnia) may lead to reductions in fatigue and improvements in energy, psychiatric wellbeing, and functioning. Similarly, research has shown that headaches and migraines are common in adults with celiac disease and can persist despite GFD adherence [48, 49]. Greater persistent headache appears to relate to greater psychiatric symptoms and role limitations.

Physical pain was endorsed to varying degrees across profiles, likely reflecting the high prevalence of bone and joint pain in celiac disease [50]. Though single-item physical pain ratings varied between profiles, there were no profile differences on SF-36 *bodily pain* or PROMIS-29 *pain interference*. Given the known relationship between chronic pain, depression and anxiety, and lower quality of life [51], some adults with celiac disease may benefit from adjunctive behavioral or medical intervention for managing headache and pain [52]. Finally, additional research is needed to operationalize the experience of food cravings in adults with celiac disease and their impact on eating behavior and quality of life [53].

Despite Profile 3’s moderate symptom burden and low subjective health as reported on the CSI, Profile 3 reported the lowest psychiatric symptoms and highest quality of life on standardized measures. Specifically, Profile 3 reported better general health, fewer role limitations due to physical health, less fatigue, and less sleep disturbance than all other profiles, and greater emotional wellbeing and better social functioning/ability to participate in social activities than Profiles 1 and 4. This finding suggests that overall symptom burden may not relate directly to worse wellbeing. Rather, specific symptoms might relate to wellbeing in different ways, and even patients with relatively lower overall symptom burden (e.g., Profile 1) may benefit from adjunctive interdisciplinary intervention to improve long-term outcomes. Additionally, patients such as those in Profile 3 may have coping skills or resilience factors that protect against deficits in psychiatric wellbeing and quality of life. Among adults with celiac disease, coping characterized by catastrophizing, emotional-oriented coping, lower perceived ability to decrease physical symptoms, and greater perceived difficulty following a GFD have been associated with lower quality of life [23, 54, 55]. On the other hand, greater celiac-specific self-efficacy and lower risk perception have been shown to predict greater quality of life [56]. Adjunctive behavioral treatment may be used to target these characteristics to increase psychiatric health and quality of life among adults such as those in Profiles 1 and 2.

There were no profile differences in self-reported GFD adherence, suggesting that differences in symptomology patterns might be explained by other factors, such as co-occurring IBS or other conditions, refractory celiac disease, or food sensitivities [11, 15, 57]. However, because of possible bias in reporting using the CDAT, this finding is inconclusive without replication using objective measures of gluten consumption and standardized dietician interview. We also found no profile differences in celiac-specific quality of life as measured by the CD-QOL. There are no cut-offs on the CD-QOL to indicate whether the present sample had objective deficits in this domain.

Finally, there were no profile differences in current age, sex, race, age at celiac disease diagnosis, or years since celiac disease diagnosis, consistent with prior research, though findings have been mixed [7]. However, Profile 4 reported lower household income and education level than Profiles 1 and 2. Income and education level are recognized social determinants of health that might influence symptomology and subjective health through mechanisms such as access to affordable gluten-free food, healthcare, specialty physicians, social support, and concomitant risk for gluten exposure [58]. While this finding is preliminary and based only on a subsample that reported income and education information, further research is needed to explore the relationships between persistent symptoms and sociodemographic variables, especially given evidence for disparities in celiac disease diagnostic testing based on black race, coverage by public insurance [59], male sex, and older age [60], and the known relationship between food insecurity and heightened risk for gluten exposure [61].

### Strengths and limitations

To our knowledge, this is the first study to examine patterns of both gastrointestinal and extraintestinal symptoms in relation to celiac disease management and wellbeing, and the first to use LPA for this purpose. Our analysis examined a comprehensive range of potential risk factors and identified several potential intervention targets to support quality of life, and physical and mental wellbeing in adults with celiac disease. Further, this sample represents U.S. adults diagnosed with celiac disease across the lifespan with an average of six years since diagnosis, which offers insight into needs of patients beyond the initial diagnosis and follow-up period.

Despite these strengths, we acknowledge several limitations. For example, the CSI does not include all symptoms of possible interest. Assessment of gastrointestinal reflux, vomiting, and constipation may be important for ruling out various co-occurring functional gastrointestinal conditions. The present study also used a self-report measure to assess GFD adherence rather than a standardized dietetic assessment or objective measure of gluten intake (e.g., stool sampling). The CDAT assesses various aspects of gluten exposure risk but may not capture actual exposure. Future research should use standardized and objective measures that are less subject to reporting biases. Additionally, the present study selected participants who reported a diagnosis of celiac disease made by biopsy, serology, or genetic testing, which introduces the possibility of false diagnosis. Celiac disease is diagnosed in those with genetic predisposition when serology identifies elevated anti-tTG, anti-endomysium, and deamidated gliadin peptide antibodies, and/or histology finds evidence of duodenal villous atrophy, intraepithelial lymphocytosis, and crypt hyperplasia. Thus, the genotype HLA-DQ2 or HLA-DQ8 is a necessary but insufficient condition for diagnosing celiac disease, and best clinical practice is to make a diagnosis only after intestinal biopsy is performed. In clinical practice however, many individuals with celiac disease may not have undergone a biopsy or genetic testing due to various reasons, such as medical cost, accessibility, or patient preferences. We aimed to reflect the diversity of individuals living with celiac disease in real-world settings and to ensure that our study was inclusive and representative of a wide range of celiac patients, considering the heterogeneity in diagnostic pathways. Future studies may consider limiting analyses to the subset of individuals who reported a biopsy-confirmed diagnosis only.

Furthermore, participants in the present study were self-selected and represent a population with access to the internet, willingness to participate in research, and capacity to complete online questionnaires. Findings may not generalize to individuals with lower socioeconomic resources or those in otherwise marginalized groups. Additionally, higher base rates of persistent symptoms and quality of life concerns may be present in our sample given that individuals with those concerns may be more likely to seek online support and more likely to contribute information to the iCureCeliac® registry, a data gathering tool for researchers seeking to improve patient outcomes in celiac disease.

Most participants in the current study identified as female and non-Hispanic white, which reflects characteristics of the diagnosed U.S. patient population [2, 4, 62–64] but may not generalize to other patient groups in the U.S. [65] and abroad. The small size of Profile 4 may reflect the sociodemographic heterogeneity of the present sample, and findings should be replicated in a more racial-, ethnic-, socioeconomic-, and gender-diverse sample.

## Conclusions

The prevalence and severity of persistent gastrointestinal and extraintestinal symptoms differ among adults with celiac disease. This study identified subgroups based on persistent symptomology, which differed in psychiatric wellbeing, functioning, and quality of life. Results suggest that lower overall symptom burden does not necessarily relate to better quality of life, and the relationship between persistent symptoms and wellbeing may be nuanced and depend on the specific symptoms and domain of quality of life assessed. Even patients with relatively low gastrointestinal symptom burden may nevertheless benefit from adjunctive treatment to address fatigue, pain, and headache, while those with other symptom profiles may not require the same. Additionally, coping skills may protect patients with greater gastrointestinal symptoms from negative quality of life outcomes. Future research should examine patterns of persistent symptoms that include a wider range of symptoms, use histological assessment and an objective measure of gluten intake to explore these relationships more robustly, assess both risk and resilience factors, and sample for sociodemographically diverse samples. This research will inform and improve healthcare for adults with celiac disease, serving to help identify patients most in need of additional support to optimize physical health and quality of life.

### Electronic supplementary material

Below is the link to the electronic supplementary material.

Supplementary Material 1

## Data Availability

The data that support the findings of this study are available from Celiac Disease Foundation but restrictions apply to the availability of these data, which were used under license for the current study, and so are not publicly available. Data are however available from the first author (Cara Dochat) upon reasonable request and with permission of Celiac Disease Foundation.

## List of Abbreviations

GFD: Gluten-free diet
IBS: Irritable bowel syndrome
CSI: Celiac Symptom Index
CDAT: Celiac Dietary Adherence Test
SF-36: RAND 36-Item Health Survey version 1.0
CD-QOL: Celiac Disease Quality of Life Survey
LPA: Latent profile analysis
BLRT: Bootstrapped likelihood ratio test
LMRT: Lo-Mendell-Rubin adjusted likelihood ratio test
AIC: Akaike Information Criterion
BIC: Bayesian Information Criterion
s-BIC: Sample size-adjusted Bayesian Information Criterion
