# Supplementary material for: Celiac disease symptom profiles and their relationship to gluten-free diet adherence, mental health, and quality of life
Source: BMC Gastroenterol. 2024 Jan 2;24:9. doi: 10.1186/s12876-023-03101-x (PMC10759532; doi:10.1186/s12876-023-03101-x)
Supplement: Supplementary file 1 — Supplementary Material 1 [file 12876_2023_3101_MOESM1_ESM.pdf]

Supplement Table 1. *Full Results of Auxiliary Analyses* (N = 317 unless otherwise specified)

**Domain**<br>Measure	Profile 1 (*n* = 117)<br>*M* (*SD*)	Profile 2 (*n* = 106)<br>*M* (*SD*)	Profile 3 (*n* = 75)<br>*M* (*SD*)	Profile 4 (*n* = 19)<br>*M* (*SD*)	Test statistic	Significant post-hoc differences, $p < .05$
**Sociodemographic Variables**						
Age	42.26 (1.43)	40.02 (1.42)	41.28 (1.78)	37.95 (3.15)	χ^2 (3,317) = 2.22, p = .53	--
Sex	Male = 0.14<br>Female = 0.85<br>Other = 0.01	Male = 0.08<br>Female = 0.91<br>Other = 0.02	Male = 0.14<br>Female = 0.86<br>Other = 0.00	Male = 0.09<br>Female = 0.91<br>Other = 0.00	χ^2 (6,317) = 5.35, p = .50	--
Race (White, Other)	White = .87<br>Other = .13	White = .95<br>Other = .05	White = .95<br>Other = .05	White = .95<br>Other = .05	χ^2 (3,317) = 4.14, p = .25	--
Education level (*n* = 278)	No school = .01<br>Some primary school = .05<br>HS diploma = .15<br>Vocational, Associates Degree, Trade School = .10<br>Some college = .42<br>Bachelors = .04<br>Masters = .19<br>Doctorate = .05	No school = .01<br>Some primary school = .03<br>HS diploma = .15<br>Vocational, Associates Degree, Trade School = .12<br>Some college = .40<br>Bachelors = .03<br>Masters = .20<br>Doctorate = .07	No school = .02<br>Some primary school = .04<br>HS diploma = .13<br>Vocational, Associates Degree, Trade School = .25<br>Some college = .32<br>Bachelors = .02<br>Masters = .19<br>Doctorate = .04	No school = .00<br>Some primary school = .07<br>HS diploma = .12<br>Vocational, Associates Degree, Trade School = .34<br>Some college = .42<br>Bachelors = .00<br>Masters = .07<br>Doctorate = .00	**χ^2 (21,277) = 45.05, p = .002**	1 v. 4<br>2 v. 4
Household income level (*n* = 228)	Less than $50K = .22<br>$50K-100K = .38<br>$100K-200K = .35<br>$200K or more = .10	Less than $50K = .24<br>$50K-100K = .36<br>$100K-200K = .26<br>$200K or more = .14	Less than $50K = .24<br>$50K-100K = .41<br>$100K-200K = .29<br>$200K or more = .06	Less than $50K = .31<br>$50K-100K = .45<br>$100K-200K = .24<br>$200K or more = .00	**χ^2 (9,277) = 36.45, p < .001**	1 v. 4<br>2 v. 4
**Disease Factors**						
Age at diagnosis	35.77 (1.41)	34.30 (1.43)	35.78 (1.78)	31.94 (3.22)	χ^2 (3,317) = 1.64, p = .65	--
Years since diagnosis	6.31 (0.77)	5.71 (0.67)	5.50 (0.78)	6.13 (1.74)	χ^2 (3,317) = 0.64, p = .89	--
Reason for diagnostic testing	Probabilities<br>Symptomatic = .74<br>Other = .26	Probabilities<br>Symptomatic = .77<br>Other = .23	Probabilities<br>Symptomatic = .80<br>Other = .21	Probabilities<br>Symptomatic = .79<br>Other = .21	χ^2 (3,317) = 0.74, p = .86	--

**Gluten-Free Diet Adherence**						
CDAT total score	13.41 (0.33)	13.67 (0.24)	12.05 (0.37)	15.18 (0.89)	**χ^2 (3,317) = 17.04, *p* = .001**	1,2,4 > 3
CDAT without symptom items	7.68 (0.23)	7.74 (0.24)	7.68 (0.29)	7.81 (0.57)	χ^2 (3,317) = 0.07, *p* = .99	--
**Anxiety and Depression**						
PROMIS-29 Anxiety	55.22 (0.89)	53.85 (0.91)	52.87 (1.12)	58.35 (2.07)	χ^2 (3,317) = 6.72, *p* = .08	4 > 2,3
PROMIS-29 Depression	52.89 (0.89)	51.28 (0.89)	50.04 (1.07)	56.96 (2.04)	**χ^2 (3,317) = 10.83, *p* = .001**	4 > 2,3
**General Health-Related Quality of Life and Functioning**						
SF-36 Physical Functioning	80.37 (2.14)	81.80 (2.10)	85.08 (2.28)	71.88 (6.11)	χ^2 (3,317) = 5.14, *p* = .16	3 > 4
SF-36 Role Limitations due to Physical Health	53.72 (4.01)	52.71 (4.10)	66.97 (4.74)	40.97 (9.20)	**χ^2 (3,317) = 9.00, *p* = .03**	3 > 1,2,4
SF-36 Role Limitations due to Emotional Health	51.36 (3.98)	61.50 (3.98)	61.82 (4.88)	31.63 (8.49)	**χ^2 (3,317) = 13.47, *p* = .004**	1,2,3 > 4
SF-36 Emotional Wellbeing	61.20 (1.85)	65.63 (1.81)	69.65 (2.13)	50.73 (4.50)	**χ^2 (3,317) = 18.71, *p* < .001**	1,2,3 > 4<br>3 > 1
SF-36 Energy/Fatigue	38.37 (2.20)	38.17 (2.25)	45.02 (2.87)	23.69 (4.16)	**χ^2 (3,317) = 18.24, *p* < .001**	1,2,3 > 4
SF-36 Social Functioning	68.14 (2.47)	72.08 (2.43)	76.33 (2.81)	60.26 (6.03)	**χ^2 (3,317) = 8.27, *p* = .04**	3 > 1,4
SF-36 Bodily Pain	61.88 (2.29)	59.32 (2.40)	65.15 (2.82)	52.68 (5.73)	χ^2 (3,317) = 4.87, *p* = .18	3 > 4
SF-36 General Health	49.72 (2.20)	49.93 (2.27)	57.36 (2.71)	42.84 (5.14)	**χ^2 (3,317) = 8.59, *p* = .04**	3 > 1,2,4
PROMIS-29 Pain Interference	52.69 (0.87)	52.80 (0.90)	51.55 (1.07)	53.37 (2.10)	χ^2 (3,317) = 1.13, *p* = .77	--

PROMIS-29 Physical Function	49.11 (0.77)	48.83 (0.79)	50.94 (0.90)	45.60 (1.88)	χ^2 (3,317) = 7.60, *p* = .06	3 > 4
PROMIS-29 Ability to Participate in Social Roles/Activities	48.79 (0.87)	50.51 (0.90)	51.88 (1.11)	44.04 (1.93)	**χ^2 (3,317) = 14.40, *p* = .002**	1,2,3 > 4<br>3 > 1
PROMIS-29 Fatigue	58.09 (1.04)	58.43 (1.07)	54.61 (1.36)	63.28 (2.2)	**χ^2 (3,317) = 12.11, *p* = .007**	4 > 1,2 > 3
PROMIS-29 Sleep Disturbance	53.76 (0.75)	52.91 (0.77)	50.20 (0.95)	57.99 (1.76)	**χ^2 (3,317) = 17.70, *p* = .001**	4 > 1,2 > 3
**Coeliac Disease-Specific Quality of Life**						
CD-QOL Total	62.72 (1.51)	62.62 (1.54)	60.59 (1.89)	65.80 (3.56)	χ^2 (3,317) = 1.90, *p* = .59	--
CD-QOL Limitations	29.72 (0.77)	29.84 (0.79)	28.94 (0.97)	31.76 (1.79)	χ^2 (3,317) = 1.97, *p* = .58	--
CD-QOL Dysphoria	9.50 (0.38)	9.08 (0.38)	8.87 (0.45)	10.22 (0.96)	χ^2 (3,317) = 2.39, *p* = .50	--
CD-QOL Health concerns	16.66 (0.46)	17.13 (0.46)	16.24 (0.58)	17.38 (1.05)	χ^2 (3,317) = 1.84, *p* = .61	--
CD-QOL Inadequate treatment	6.85 (0.19)	6.58 (0.20)	6.55 (0.24)	6.40 (0.46)	χ^2 (3,317) = 1.68, *p* = .64	--

Note. *M* = mean; *SD* = standard deviation; CSI = Celiac Symptom Index; CDAT = Celiac Dietary Adherence Test; PROMIS = Patient-Reported Outcomes Measurement Information System®; SF-36 = Short-Form 36; CD-QOL = Coeliac Disease Quality of Life Survey. All values are raw scores except for PROMIS measures**,** which are *t*-scores. **Bold** text indicates $p \leq .05$.
